# Case Report: Dynamic Changes in Hemodynamics During the Formation and Progression of Intracranial Aneurysms

**DOI:** 10.3389/fcvm.2021.775536

**Published:** 2022-01-21

**Authors:** Xiaodong Zhai, Yadong Wang, Gang Fang, Peng Hu, Hongqi Zhang, Chengcheng Zhu

**Affiliations:** ^1^Department of Neurosurgery, Xuanwu Hospital, Capital Medical University, Beijing, China; ^2^China International Neuroscience Institute (China-INI), Beijing, China; ^3^Department of Neurosurgery, Weihai Municipal Hospital, Weihai, China; ^4^Department of R&D, UnionStrong (Beijing) Technology Co. Ltd., Beijing, China; ^5^Department of Radiology, University of Washington School of Medicine, Seattle, WA, United States

**Keywords:** intracranial aneurysms, natural history, hemodynamic analysis, dynamic changes, computational fluid dynamics

## Abstract

Despite the devastating consequences of aneurysmal subarachnoid hemorrhage (SAH), the mechanisms underlying the formation, progression, and rupture of intracranial aneurysms (IAs) are complex and not yet fully clear. In a real-world situation, continuously observing the process of aneurysm development in humans appears unrealistic, which also present challenges for the understanding of the underlying mechanism. We reported the relatively complete course of IA development in two real patients. On this basis, computational fluid dynamics simulation (CFD) was performed to evaluate the changes in hemodynamics and analyze the mechanism underlying the formation, progression, and rupture of IAs. Our results suggested that the formation and progression of IAs can be a dynamic process, with constantly changing hemodynamic characteristics. CFD analysis based on medical imaging provides the opportunity to study the hemodynamic conditions over time. From these two rare cases, we found that concentrated high-velocity inflow jets, flows with vortex structures, extremely high WSS, and a very steep WSSG were correlated with the formation of IAs. Complex multi-vortex flows are possibly related to IAs prior to growth, and the rupture of IAs is possibly related to low WSS, extreme instability and complexity of flow patterns. Our findings provide unique insight into the theoretical hemodynamic mechanism underlying the formation and progression of IAs. Given the small sample size the findings of this study have to be considered preliminary and exploratory.

## Introduction

Intracranial aneurysms (IAs) are a common neurovascular disease with an estimated prevalence of 3–5% in the general population (1–3). Most cases of non-traumatic subarachnoid hemorrhage (SAH), which is a devastating condition with high rates of mortality and morbidity, is caused by the rupture of IAs (4–6). The natural history of aneurysmal formation, growth, and eventual rupture are thought to be closely related to the progressive degradation of the vascular wall in response to abnormal hemodynamics, especially, the wall shear stress (WSS) computed by computational fluid dynamics (CFD) analysis (7–10). Abnormally low or high WSS at aneurysm regions are both commonly seen on explanation of evolution of IAs (8, 11).

In a real-world situation, it is extremely rare for imaging examinations to capture the continuous progression from normal cerebral arteries to aneurysmal formation and subsequent growth and rupture in humans. It is rare to have 3D imaging of the normal artery before the development of intracranial aneurysm, and then such an aneurysm ruptured during imaging follow up. Therefore, most of the explorations can only focus on a certain stage in the development of IAs, such as the morphological or hemodynamic analysis of the rupture risk of IAs (10, 12–15). In addition, there are differences in hemodynamic analysis methods and CFD modeling used in different studies, which leads to poor comparability between the quantitative results of hemodynamics. Thus, currently, the hemodynamic mechanism of the formation, progression and rupture of IAs is not conclusively understood, and contradictory findings are frequently seen.

In this study, we reported a relatively complete progression from normal intracranial arteries to aneurysmal formation, growth, and even rupture in real patients. Furthermore, we performed hemodynamic analysis on these patients with the aim of assessing the changes in hemodynamics to provide a theoretical basis for the underlying mechanism of the formation and progression of IAs.

## Case Description

**Figure 1:**
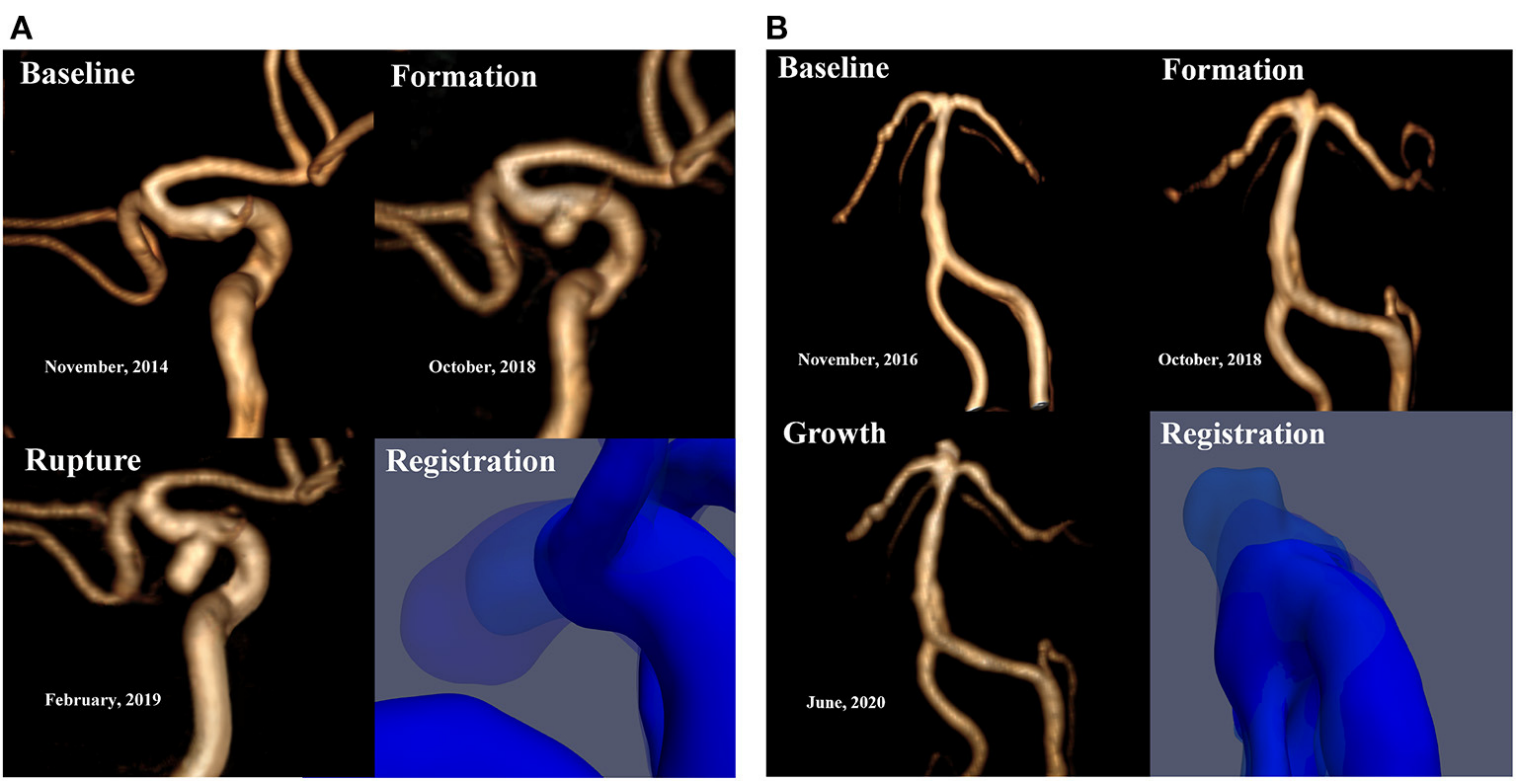
**(A)** Patient 1 is a 56-year-old female. No significant abnormalities were observed by the baseline MRA. MRA reexamination about 4 years later showed a newly formed saccular aneurysm with a diameter of 3.2 mm in the left posterior communicating artery. CTA showed that the aneurysm had significantly increased in size, reaching a diameter of 6.9 mm. **(B)** Patient 2 is a 47-year-old female. No significant abnormalities were observed by the baseline MRA. A newly formed basilar tip aneurysm and aneurysmal enlargement were observed in the subsequent MRA examinations at follow-up (from 2.1 to 3.8 mm in diameter). Registration of 3D surfaces with different degrees of transparency showed changes on continuous follow-up angiography.

Here, we report two patients. (1) The first patient was a 56-year-old female with a history of hypertension and coronary heart disease who presented with a 10-day history of limb weakness (November 18, 2014). Cranial magnetic resonance imaging (MRI) and magnetic resonance angiography (MRA) assessments did not reveal obvious abnormalities (Figure 1A, Baseline), and the patient's symptoms resolved spontaneously without any interventions. MRA reexamination of the patient 4 years later (October 9, 2018) showed a newly formed saccular aneurysm with a diameter of 3.2 mm in the left posterior communicating artery (Figure 1A, Formation). Nevertheless, due to the small size of the aneurysm, the patient opted for follow-up observation. She was diagnosed with acute SAH by cranial computed tomography (CT) performed in response to a sudden headache 4 months later (February 14, 2019). CT angiography (CTA) showed that the aneurysm had significantly increased in size, reaching a diameter of 6.9 mm (Figure 1A, Rupture). Microsurgical clipping of the aneurysm was immediately performed, and the patient recovered well in the ward postoperatively. (2) The second patient shared some similarities with the first in terms of the developmental process of the aneurysm. Patient 2 was a 47-year-old female who underwent MRA assessment due to dizziness on November 13, 2016; the assessment did not detect any obvious abnormalities. A newly formed basilar tip aneurysm and aneurysmal enlargement were observed in the follow-up MRA examinations performed on October 9, 2018 (Figure 1B, Formation), and June 7, 2020 (Figure 1B, Growth; from 2.1 to 3.8 mm in diameter), respectively. The patient eventually received successful endovascular treatment after aneurysmal enlargement occurred.

## Image Acquisition and Reconstruction

For this study, all the three-dimensional (3D) TOF- MRA were performed on the same 3.0 T Siemens scanner (Erlangen, Germany) in the same department except the Patient 1 had undergone 3D CTA at the third follow-up due to the acute rupture of aneurysm. Examinations for TOF-MRA were performed with the following parameters: TR, 20 ms; TE, 3.6 ms; slice thickness, 0.7 mm; field of view, 210 mm; flip angle, 18°; number of slices, 140; total acquisition time, 3:17 min; and voxel size, 0.3 × 0.3 × 0.7 mm.

Growth of aneurysm was defined as a size increase of at least 1.0 mm in any dimension on angiographic or cross-sectional imaging (16). The tomographic data and the morphological parameters of the enrolled patients were recorded based on the Computer-Assisted Semi-Automated Measurement (CASAM) (17). CFD analysis was applied to assess these vessels and aneurysms to assess the differences in hemodynamics.

The methods used for image reconstruction and CFD simulation of hemodynamic studies were the same as described in our previous publications (18–21). CFD results are depended on the quality of image segmentation process. To maintain the consistency of the models within the same patient, we performed image registration and fusion *via* 3D Slicer (version 4.5.0), then an iterated select threshold value was applied for image segmentation to the same patient. In the subsequent thresholding step, we used the Mimics Medical software (Version 19.0, Materialize, Leuven, Belgium) to segment the models in order to preserve the length and shape of the vessel, while only the aneurysm or region of aneurysm formation was different (22). In the end, we saved the segmented surface geometry in the Standard Tessellation Language format as the input for the next step.

## CFD Modeling and Hemodynamics Analysis

Each 3D aneurysm model was subdivided into aneurysm sac and parent artery regions before meshing. Then, each 3D model geometry was imported into the ICEM CFD software (ANSYS Inc., Canonsburg, Pennsylvania, USA). Different mesh sizes we set for different parts. For the inlet, outlet, and sac part, we set 0.1 mm that meets most aneurysms' size, and 0.3 mm was set for the parent artery part. The total of finite volume tetrahedral element grids are approximately 1 million with four layers of prism elements for CFD simulations.

After meshing, ANSYS CFX 18.0 (ANSYS Inc.) was then used for hemodynamic simulation. A Newtonian fluid was assumed, and a density of 1,060 kg/m^3^ and a dynamic viscosity of 0.0035 N s/m^2^ were modeled. The vessel wall was assumed to be rigid with a no-slip boundary. Because the patient-specific boundary conditions were not available, the inflow boundary condition was a representative pulsatile velocity profile obtained from the averaged normal human (23). A traction-free boundary condition was applied to all outlets (24). Initial pressure and velocity were set to zero. Three cardiac cycles were simulated to minimize transient numerical errors. Results from the third simulated cardiac cycle were collected as output for the final analyses. Validation of these methods (consistency, reliability) has been demonstrated in our previous publication (18, 19).

To analyze the hemodynamics changes that occur during the formation and progression of IAs, vital hemodynamic parameters including WSS, wall shear stress gradient (WSSG), pressure and energy loss (EL) were calculated based on the simulated pulsatile flow simulations (18, 19). Normalized wall shear stress (NWSS) and normalized pressure (NP), defined as the WSS and pressure of the aneurysm wall divided by that of the parent artery wall, respectively, were calculated (25, 26). The region of aneurysmal formation was marked by the registration technique through comparative cross-sectional imaging before and after aneurysm formation.

## Results

**Figure 2:**
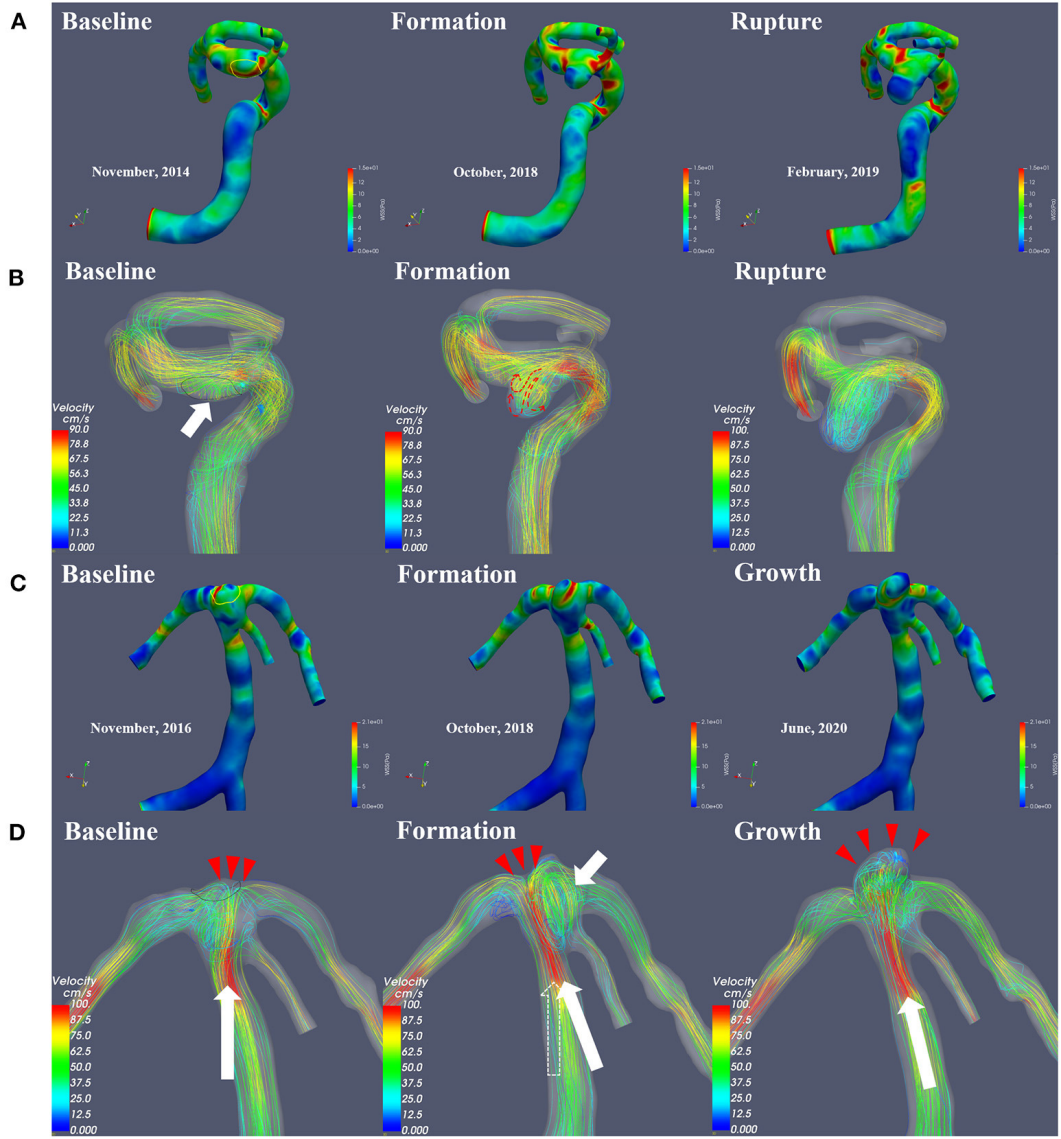
This figure illustrates the change in hemodynamic parameters during the formation, progression and rupture of IAs. The WSS distribution **(A,C)** and flow velocity profile **(B,D)** were shown, respectively. The area within the yellow line is the region of aneurysmal formation marked by the registration technique. Prior to aneurysm formation, the regions feature flows with vortex structures (**B**, short white arrow). In the impact region (**D**, Baseline, red arrows) of the concentrated high-velocity inflow jet (**D**, Baseline, long white arrow indicates blood flow direction), a newly formed basilar tip aneurysm was visible on follow-up angiography **(D)**. Affected by vortex flow after the formation of the aneurysm (**D**, Formation, white short arrow), the main direction of flow shifted (**D**, Formation, from the original dotted arrow to the long white arrow), causing saccular dilatation in the new impact region (**D**, Formation, red arrows), and the size of the aneurysm increased (**D**, Growth). A complex multi-vortex flow is associated with future aneurysm growth, and rupture is related to extreme instability and complexity of blood flow patterns.

The qualitative hemodynamic analysis revealed that, prior to aneurysm formation, the regions featured concentrated high-velocity inflow jets (Figure 2D) or flows with vortex structures (Figure 2B). A complex multi-vortex flow is associated with aneurysms prior to growth (Figures 2B,D), and the future aneurysm rupture is related to extreme instability and complexity of blood flow patterns (Figure 2B).

**Figure 3:**
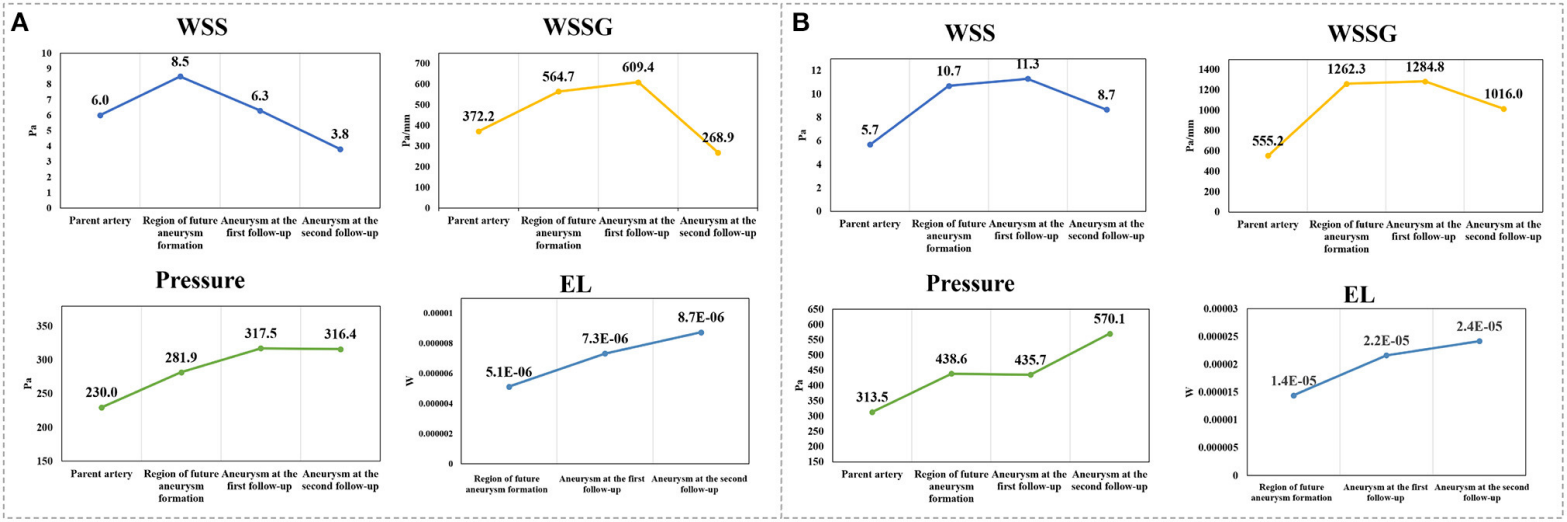
**(A,B)** Display the trends in the main hemodynamic parameters of patient 1 and patient 2, respectively, during continuous follow-up angiography. WSS, wall shear stress; WSSG, wall shear stress gradient; EL, energy loss.

As shown in Figures 2, 3, the results from quantitative hemodynamic analysis indicated that the WSS, WSSG, and pressure in the regions prior to aneurysm formation were significantly higher than those in the parent artery. Compared with the parent artery, the value of NWSS and NP of regions prior to aneurysm formation are larger. In the progression from normal vessels to aneurysmal formation, the WSSG and EL increased in both patients. As the aneurysm further grew, the WSS and WSSG decreased, and the pressure and EL increased (Patient 2, Figure 3B). As shown in Figure 3A, the WSS and WSSG of ruptured aneurysms were decreased, and the EL was increased (Patient 1, Figure 3A). The detailed numerical results from hemodynamic analysis of patients are summarized in Table 1.

**Table 1:** The hemodynamic parameters result of included patients.

**Patient no**.	**Subject**	**Mean WSS (Pa)**	**Mean NWSS**	**Mean WSSG (Pa/mm)**	**Mean pressure (Pa)**	**Mean NP**	**Energy loss (W)**
Patient 1	Parent artery	6.0	1	372.2	230.0	1	–
Patient 1	Baseline	8.5	1.4	564.7	281.9	1.2	5.12E-06
Patient 1	Formation	6.3	0.9	609.4	317.5	1.2	7.33E-06
Patient 1	Rupture	3.8	0.6	268.9	316.4	1.1	8.74E-06
Patient 2	Parent artery	5.7	1	555.2	313.5	1	–
Patient 2	Baseline	10.7	1.9	1,262.3	438.6	1.4	1.44E-05
Patient 2	Formation	11.3	2.0	1,284.8	435.7	1.4	2.17E-05
Patient 2	Growth	8.7	1.4	1,016.0	570.1	1.3	2.42E-05

*WSS, wall shear stress; WSSG, wall shear stress gradient; NWSS, normalized wall shear stress; NP, normalized pressure*.

## Discussion

In this study, we reported a relatively complete progression from normal intracranial arteries to aneurysmal formation and growth in two real patients. Patient 1 experienced even rupture at the third follow-up. Furthermore, we investigated the changes in hemodynamics with the aim of providing a theoretical basis for the underlying mechanism of the formation and progression of IAs. The results of this study indicated that the formation and progression of IAs can be a dynamic process, with constantly changing hemodynamic characteristics.

Very few subjects had prophylactic cerebrovascular imaging examination prior to the formation of an aneurysm; thus, previous studies assessing the hemodynamic characteristics in the regions before aneurysmal formation usually did so after virtually removing the aneurysms and reconstructing the parent artery, thereby restoring an image of the normal artery before aneurysmal formation (27–29). However, the geometry of the parent artery can change after the aneurysm formation. In the present study, the results from real patients revealed that the regions of future aneurysm formation featured concentrated high-velocity inflow jets or flows with vortex structures. Consistent with findings from previous virtual studies (14, 30), the WSS, WSSG, and pressure at the future aneurysm sites were significantly higher than those in the parent artery. Narrowing proximal vessel leads to flow acceleration that accentuates WSS and spatial gradients at the bifurcation apex of Patient 2, large branch angles and proximal parent vessel tapering may increase the risk of IA formation (31, 32).

The contribution of hemodynamics to the progression of IAs is complex and controversial, and both high-WSS and low-WSS theories have been proposed to explain the growth and rupture of IAs (8, 33–35). The hemodynamic results of natural history data revealed that the hemodynamic parameters of IAs have constantly changed with the development of the aneurysm, and increasing or decreasing tendency presented in different stages of development. It implies that the formation and progression of IAs could be a dynamic process in which the hemodynamic characteristics are constantly changing. The complex interactions of hemodynamics in the progression of IAs are difficult to accommodate within high-WSS, low-WSS, or any other single existing theories. In light of previous work and our present results, we inferred that in the early stages of aneurysm formation and development, the WSS and WSSG of the aneurysm, which are higher than those of the parent artery, may continue to increase in the short term. The hemodynamic energy of the blood flowing through the aneurysm sac acts on the aneurysm wall. As the size of the aneurysm continues to grow, the energy loss of the blood flow through the aneurysm gradually increases, and the WSS and WSSG subsequently decrease. Low WSS was considered to be associated with aneurysm rupture (14, 36). As a result of degenerative remodeling, the aneurysm wall becomes too fragile to resist the stress of blood flow, and the aneurysm ultimately ruptures.

## Limitation

The primary limitation of this study was the small sample size, and the follow-up time varies. As the natural history data from developing IAs is very rare, the present study was only able to include two cases. Second, the image of Patient 1 after aneurysm ruptured was performed using CTA instead of MRA, which may lead to biased results. In addition, owing to the missing of patients' specific inflow conditions, the inflow boundary condition was a representative pulsatile velocity profile. But the intra-individual flow variation and the variation on subsequent hemodynamic simulations using the inlet flow as a boundary condition in serially acquired 2D phase-contrast MR data were considered relatively small for hemodynamics patterns (big for absolute values) (37). Common limitations for the hemodynamic simulations are a Newtonian fluid and have rigid walls for modeling blood vessels, which could lead to biased results. The additional limitation seems to be homeostasis, lipid status and medical treatment from the same patient over different periods, but in case of describing two cases, only the statistical impact of these clinical factors is very difficult to measure. Nevertheless, even limitations exist, this study based on rare and precious data inspires researchers for understanding the underlying hemodynamic mechanism of IAs development.

## Conclusion

The formation and progression of IAs can be a dynamic process, with constantly changing hemodynamic characteristics. CFD analysis based on medical imaging provides the opportunity to study the hemodynamic conditions over time and is a promising tool to study the pathology of intracranial aneurysm *in vivo*. Concentrated high-velocity inflow jets, flows with vortex structures, extremely high WSS, and a very steep WSSG were correlated with the formation of IAs in the two cases. Complex multi-vortex flows are possibly related to IAs prior to growth, and the rupture of IAs is possibly related to low WSS, extreme instability and complexity of flow patterns. Our findings provide unique insight into the theoretical hemodynamic mechanism underlying the formation and progression of IAs. Owing to the small sample size, the findings of this study have to be considered preliminary and exploratory.

## Data Availability Statement

The original contributions presented in the study are included in the article/supplementary material, further inquiries can be directed to the corresponding authors.

## Ethics Statement

The studies involving human participants were reviewed and approved by the Ethics Board of Xuanwu Hospital, Capital Medical University. The patients/participants provided their written informed consent to participate in this study. Written informed consent was obtained from the individual(s) for the publication of any potentially identifiable images or data included in this article.

## Author Contributions

XZ, YW, GF, PH, HZ, and CZ contributed to the conception, design, analysis, and interpretation of the data as well as to drafting the article and revising it critically. All authors have read and approved the final version of the manuscript.

## Funding

This work was supported by National Key R&D program of China with grant 2016YFC1300800, Beijing Municipal Administration of Hospitals' Ascent Plan with grant DFL20180801, and the Beijing Scientific and Technologic Project (Z201100005520021).

## Conflict of Interest

GF was employed by the company Union Strong (Beijing) Technology Co. Ltd. The remaining authors declare that the research was conducted in the absence of any commercial or financial relationships that could be construed as a potential conflict of interest.

## Publisher's Note

All claims expressed in this article are solely those of the authors and do not necessarily represent those of their affiliated organizations, or those of the publisher, the editors and the reviewers. Any product that may be evaluated in this article, or claim that may be made by its manufacturer, is not guaranteed or endorsed by the publisher.
